# A Two-Stage Network for Zero-Shot Low-Illumination Image Restoration

**DOI:** 10.3390/s23020792

**Published:** 2023-01-10

**Authors:** Hao Tang, Linfeng Fei, Hongyu Zhu, Huanjie Tao, Chao Xie

**Affiliations:** 1 College of Mechanical and Electronic Engineering, Nanjing Forestry University, Nanjing 210037, China; 2 School of Computer Science, Northwestern Polytechnical University, Xi’an 710072, China; 3 College of Landscape Architecture, Nanjing Forestry University, Nanjing 210037, China

**Keywords:** low-illumination image enhancement, zero-shot learning, Retinex theory, image feature

## Abstract

Due to the influence of poor lighting conditions and the limitations of existing imaging equipment, captured low-illumination images produce noise, artifacts, darkening, and other unpleasant visual problems. Such problems will have an adverse impact on the following high-level image understanding tasks. To overcome this, a two-stage network is proposed in this paper for better restoring low-illumination images. Specifically, instead of manipulating the raw input directly, our network first decomposes the low-illumination image into three different maps (i.e., reflectance, illumination, and feature) via a Decom-Net. During the decomposition process, only reflectance and illumination are further denoised to suppress the effect of noise, while the feature is preserved to reduce the loss of image details. Subsequently, the illumination is deeply adjusted via another well-designed subnetwork called Enhance-Net. Finally, the three restored maps are fused together to generate the final enhanced output. The entire proposed network is optimized in a zero-shot fashion using a newly introduced loss function. Experimental results demonstrate that the proposed network achieves better performance in terms of both objective evaluation and visual quality.

## 1. Introduction

In the field of image processing, low-illumination image restoration is one of the most important branches and can be used in a wide range of advanced vision tasks such as military, surveillance, and security [1,2]. However, due to the limitations in recording equipment and environmental factors, images and videos, especially when captured at night, are severely degraded and large amounts of information can be lost in high-level visual processing tasks [3,4]. Although a longer exposure time improves the image brightness to some extent, it has few practical applications [5]. The aim of low-light image enhancement is to highlight useful features of an image while weakening or eliminating noise and improving contrast to produce better visual perception for human eyes [6,7]. Researchers have proposed a large number of low-light image enhancement algorithms, and they can be roughly divided into conventional enhancement methods and deep-learning-based methods [8,9].

Conventional ones are mainly developed on the basis of image histograms [10] and Retinex theory [11]. Based on histogram equalization (HE), Zhu et al. [12] proposed local histogram equalization, which uses a sliding window to chunk the image, resulting in significant local detail enhancement compared to original HE. Since then, sliding window overlap algorithms, sliding window partial overlap algorithms, and so on have emerged successively. Retinex theory decomposes the image into a light map and a reflectance map. Earlier algorithms used the reflection map as an image enhancement effect, and the method would amplify the noise in the image, which would affect the final result. Researchers have made many improvements to address the problem of image noise, including the single scale Retinex algorithm (SSR) [13], multi-scale Retinex algorithm (MSR) [14], multi-scale Retinex algorithm with image recovery (MSRCR) [15], multi-scale Retinex algorithm based on HSV space, etc.

Deep learning has achieved good performance in the field of low-illumination image enhancement because of its powerful feature representation and non-linear mapping capabilities. Lore et al. [16] were the first to implement low-light image enhancement (LLNet) using a deep learning approach, which proposes a deep autoencoder to identify signal features from low-light images, capable of adaptively enhancing image brightness without oversaturation. The increase in the image datasets in terms of quality as well as in quantity has given rise to many Retinex-based deep learning methods that enable low-light image enhancement by better estimating the reflectance component and enhancing the luminance of illuminated images. Some of the Retinex-based deep learning methods incorporate BM3D or channel attention mechanisms as a way of reducing image noise and extracting image features to achieve better visual results [17]. Subsequent methods have been improved in terms of targeting the limitations of the dataset, as well as overfitting and real-time problems, improving the application of deep learning methods in the field of low-illumination image enhancement.

Deep-learning-based approaches are the main trend in this research direction [18]. Although some of the low-light image enhancement algorithms based on deep learning have achieved remarkable results, there are still several problems to be solved. Firstly, existing methods mostly use fully supervised learning, although the illumination estimation under such a framework is inherently ill-posed. Secondly, the enhancement effect of fully supervised learning methods is heavily influenced by their training datasets, and thus there are some common problems such as loss of image details, noise amplification, and color distortion in the enhancement effect. Therefore, we propose a low-illumination enhancement algorithm that requires only a single low-illumination image input in this paper, and our contributions are summarized as follows:

- We propose a two-stage low-illumination image restoration network, in which a pre-decomposition submodule is incorporated to divide the original image into illumination, reflectance, and feature. Moreover, the whole network is optimized in a zero-shot way instead of using supervised learning.
- To guide the decomposition network focusing on the dark area of the image, a new loss function is proposed for our network. The loss function can also obtain relatively clearer texture features in the dark area, and avoid the problem of overexposure or underexposure in the others areas.
- Experiments show that our method achieves better performance on the benchmark datasets. Compared with recent methods based on decomposition theory, the proposed method can visually better retain the detailed features of images and avoid the problem of overexposure. There is a significant improvement in *PSNR* and *SSIM* (reference evaluation indices), and *NIQE* and *LOE* (no-reference evaluation indices).

The rest of this paper is organized as follows: In Section 2, we review representative Retinex-model-based and deep-learning-based methods for low-illumination image enhancement. In Section 3, the model proposed in this paper is presented. The fourth section introduces the loss function in detail. Section 5 gives the experimental results and evaluation. The sixth part summarizes the whole paper.

## 2. Related Work

### 2.1. Retinex-Model-Based Approach

The Retinex-model-based approach enhances low-illumination images by the idea of decomposition. The principle of Retinex theory, first proposed by Land and widely used in image processing, can be expressed as S=R∗I, where ∗ denotes elemental multiplication. S is the original image, R is the reflection map, and I is the illumination map. The brightness and contrast of the image are improved by retaining the reflectance properties while adjusting the intensity of the light map. Jobson et al. [11] proposed the single scale Retinex algorithm (SSR), which uses the construction of a Gaussian surround function to filter each of the three-color channels of the image as the estimated light component, and obtains the output after subtracting the logarithmically processed original image from the light map. The multi-scale Retinex algorithm (MSR) [19] adds the number of Gaussian surround functions to the SSR and can be seen as a weighted summation of multiple SSRs that do not pass the scale. The adaptive multi-scale Retinex algorithm based on the HSV color channel first transforms the image spatially, estimating the image light component in luminance space before transferring back to RGB space [20].

### 2.2. Learning-Based Approach

In recent years, low-light image enhancement methods based on deep learning have become popular and have made good progress. Starting in 2017, a deep-encoder-based method was first proposed, which uses a variant of the stacked sparse denoising self-encoder to identify features from shimmering images, adaptively enhance and denoise them, and simultaneously highlight and denoise the images [16]. Shen et al. [21] proposed a multi-scale Retinex combined with a CNN model that learns the end-to-end mapping between low-light and bright images directly, with the network structure containing a residual structure that makes full use of the information in each convolutional layer and the relevant parameters set by backpropagation. Wang et al. [22] proposed the GLADNet network structure, which first performs global illumination estimation of low-illumination images and then reorganizes the details using the global part of the generated illumination in connection with the original input, and the connection to the input image complements the details. The aforementioned fully supervised learning methods have achieved good results in self-illuminating image enhancement, but such methods require image pairs that often need to be manually adjusted for parameters. There is still a certain gap between the manually adjusted images and the real images, which limits the generalizability of fully supervised learning methods in real environments.

Compared to fully supervised learning methods, unsupervised and zero-shot learning methods have become more popular in recent years as they effectively avoid reliance on datasets by constructing loss functions and constraining the images themselves to achieve enhancement. Jiang et al. [23] first proposed the unsupervised learning method called EnlightenGAN. EnlightenGAN is the first method to successfully introduce unpaired training into low-light image enhancement. The method creates a mapping between unpaired low-light and normal-light image domains to enable unsupervised training. Fu et al. [24] proposed the low-illumination enhancement network (LE-GAN) using an identity invariant loss and attention module, which enhances the feature extraction of images using an illumination-aware attention module to improve visual quality while achieving noise reduction and detail enhancement. In addition, identity-invariant loss can solve the overexposure problem. Zhang et al. [25] trained a small CNN network (ExCNet), where the network does not need to be trained in advance and then tested. It can estimate the best-fit s-curve directly for a given backlit image. With its s-curve, the backlit image can be recovered accordingly. These unsupervised learning methods have improved considerably in terms of generalization ability, but there is still much to be achieved in terms of image brightness, contrast, and color bias.

Compared with the above methods, the proposed method integrates image smoothing into the decomposition network to extract the global features of the image and avoid the loss of image details caused by the smoothing operation. This method can accurately estimate the reflectance map, illumination map, and feature map of the image. Moreover, this method does not need pairwise image pairs for training, which ensures its ability to generalize to the illumination environment.

## 3. Methodology

**Figure 1:**
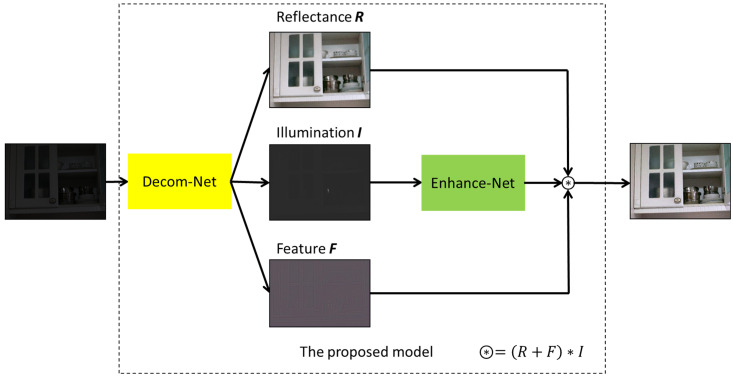
The framework of the proposed model.

In order to better preserve image details and improve image brightness at the same time, as shown in Figure 1, the method in this paper is divided into two parts: decomposition and enhancement. In the decomposition part, the image is first decomposed into illumination map, reflection map, and feature map. Then the illumination map is separately enhanced, and finally the final effect is obtained by adaptive fusion. The remainder of this section describes both modules in detail.

### 3.1. Decom-Net

**Figure 2:**
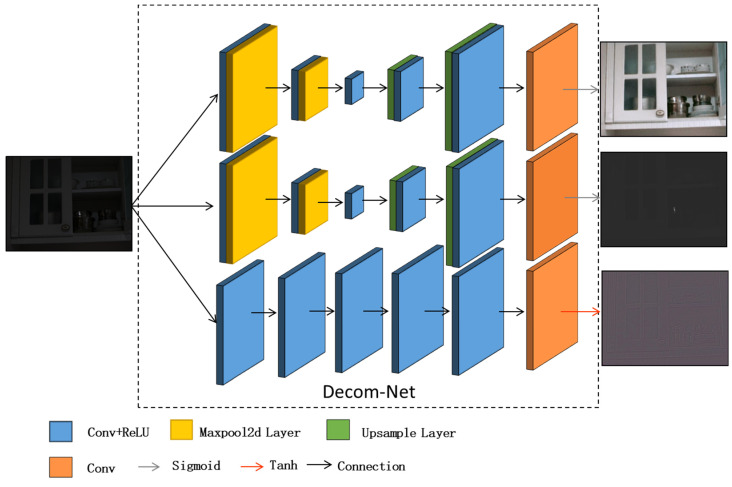
The model structure diagram of the Decom-Net.

Our Decom-Net is inspired by the Retinex theory. However, the original RetinexNet proposed by Chen et al. [17] erases many detailed features of the image when noise reduction is applied to the image. In response, we propose a three-branch convolutional decomposition network, shown in Figure 2, with three branches for estimating the illumination map, the reflectance map, and the feature map, respectively. It features a pooling layer in the decomposition process, which allows the illumination and reflectance maps to be denoised during the decomposition process. The Sigmoid function is chosen for the activation functions of the illumination and reflectance maps to ensure that the output is between 0 and 1. The activation function for the feature map is chosen as the tanh function. The output of tanh is between −1 and 1, which has faster model convergence. The decomposition of the image is carried out when the number of iterations has been reached and the loss function has reached its minimum value.

### 3.2. Enhance-Net

**Figure 3:**
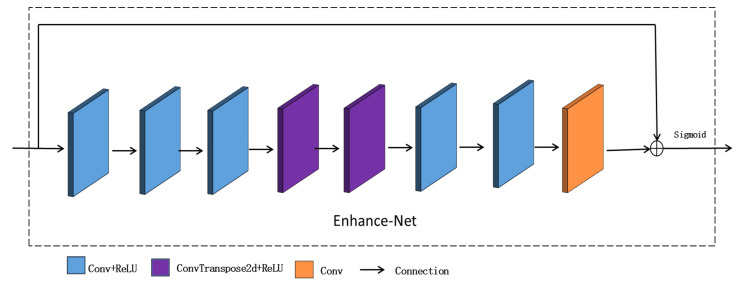
The model structure diagram of the Enhance-Net.

The main purpose of the Enhance-Net is to adjust the brightness of the illumination, as shown in Figure 3. The input of the module is the illumination map output by the Decom-Net. The module is composed of eight convolutional layers, which can effectively obtain the illumination information of the illumination map. In order to make up for the possible loss of the effective information of the illumination map during the process, the input layer is finally spliced to the last layer, and the output is the adjusted illumination map.

## 4. Loss Function

We decompose low-illumination image *S* into three parts: illumination map *I*, reflectance map *R*, and feature map *F*.

(1) $$S=\left(R+F\right)*I$$

To better configure the network weights, we set up a loss function to guide the current network to generate a more accurate branching section. This loss function is as follows:

(2) $$L=L_{recon}+\lambda_{1}L_{s}+\lambda_{2}L_{t}$$

where $L_{recon}$ is the reconstruction loss function, $L_{s}$ is the smoothness loss, and $L_{t}$ is the feature estimation loss. $\lambda_{1}$ and $\lambda_{2}$ are the weight factors.

### 4.1. Reconstruction Loss

In Retinex theory, the channel maxima of *R*, *G*, and *B* are usually used as an initial estimate of illumination, and the reflectance image is obtained by pixel segmentation between the original image and the illumination map. Moreover, we assume that the three-color channels have the same illumination. Here, we follow the idea of Retinex theory as a constraint on reflectance and illumination. The reconstruction losses in this paper are as follows:

(3) $$L_{recon}=\|S-\tilde{S}{\|}_{1}+\|I-\tilde{I}{\|}_{1}+\|R-\tilde{R}{\|}_{1}$$

where S represents the input image, $\tilde{S}$ is the reconstructed image, I is the decomposed illumination map, and $\tilde{I}$ is the color channel maximum. R is the decomposed reflectance map, $\tilde{R}=S/I$, and $L_{1}$-norm is used to bootstrap all loss functions in this paper.

### 4.2. Smoothness Loss

In this paper, in terms of noise, the reflectance and illumination maps should be properly denoised to avoid the amplified noise on the enhancement effect. The loss function is as follows:

(4) $$L_{s}=\frac{1}{HWC}\overset{H}{\underset{i=1}{\sum }}\overset{W}{\underset{w=1}{\sum }}\overset{C}{\underset{c=1}{\sum }}\left(\left\|\Delta_{x}\tilde{S}\|+\|\Delta_{y}\tilde{S}\right\|\right)$$

where *H*, *W*, and *C*, respectively, represent the height, width, and channel of the image.

### 4.3. Feature Estimation Loss

In a low-illumination image enhancement task, it is inevitable to lose some detail features. In the RetinexNet and other methods, smoothing reduces the sharpness of the image itself, and the image becomes blurred. Therefore, it is necessary to extract the image features separately in the network using the smoothing operation. In this paper, weighted guide image features are extracted according to the estimated illumination map. The loss function is as follows:

(5) $$L_{t}=\|S\cdot F{\|}_{F}+\frac{\left[\|\beta \cdot {\left(\alpha_{x}R\right)}^{2}{\|}_{1}+\|\beta \cdot {\left(\alpha_{y}R\right)}^{2}{\|}_{1}\right]}{\lambda_{2}}$$

where $\|\;{\|}_{F}$ represents the Frobenius norm of the matrix, β is the illumination guidance weight, and its expression is as follows:

(6) $$\beta =normalize{\left[I\cdot {\left(\alpha_{x}R\right)}^{2}\cdot {\left(\alpha_{y}R\right)}^{2}\right]}^{-1}$$

where *normalize* denotes min-max normalization.

## 5. Experimental Results and Analysis

In all experiments, we empirically set $\lambda_{1}$ = 0.5 and $\lambda_{2}$ = 5000. All experiments in this article were conducted in the same configuration environment, the training environment configuration: Intel I7-8700 CPU, 32 GB RAM, and NVIDIA GeForce RTX2080 Ti GPU. PyTorch framework, PyCharm software in 32 GB environment, and Anaconda Python 3.7 interpreter built the network framework. For the sake of fairness, two low-light image datasets, LOL [17] and 5 K [26], were selected for comparison. The methods selected for comparison include HE [10], Retinex [19], ExCNet [25], RRDNet [27], LightenNet [28], Zero-DCE [29], DSLR [30], and LLNet [16], and the results of all comparison methods are reproduced from their official code.

### 5.1. Subjective Evaluation

**Figure 4:**
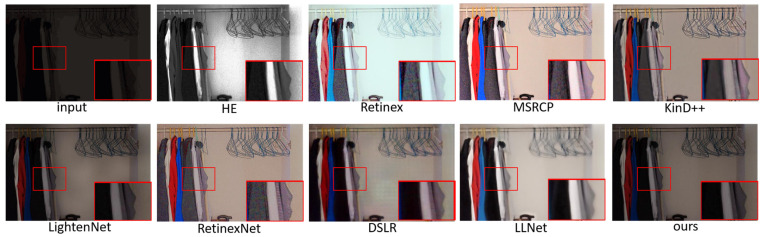
Visual comparison of enhanced renderings of low-illumination images.

**Figure 5:**
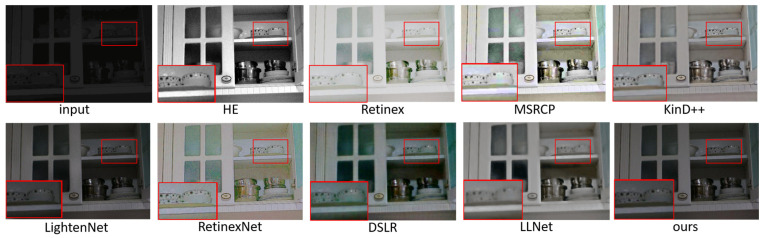
Visual comparison of enhanced renderings of low-illumination images.

**Figure 6:**
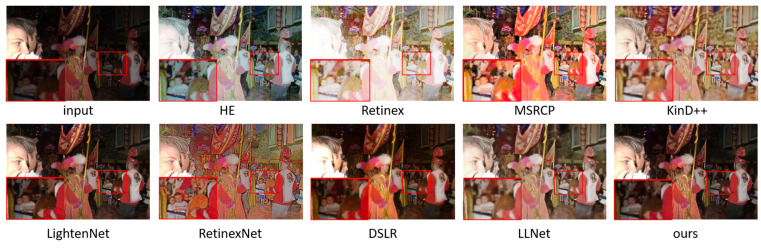
Visual comparison of enhanced renderings of low-illumination images.

**Figure 7:**
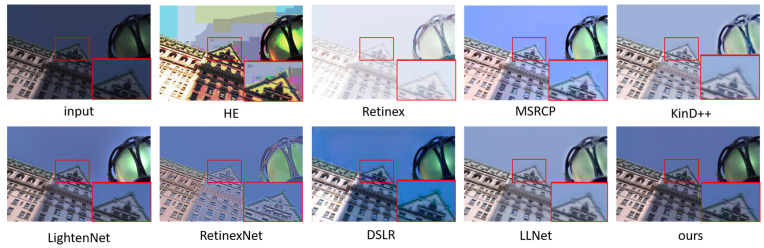
Visual comparison of enhanced renderings of low-illumination images.

We show the enhancement effects of various algorithms in Figure 4, Figure 5, Figure 6 and Figure 7, and enlarge some of the details to better carry out subjective visual evaluation. Figure 4 and Figure 5 belong to the LOL dataset. In Figure 4, HE significantly improves the image brightness by pulling up the contrast, but the overall image distortion is serious. Retinex has the best visual brightness improvement effect in Figure 4, but the clothing color is distorted and obvious noise can be seen in the enlarged image. Compared with the previous two methods, ExCNet avoids the impact of color distortion, but the detail is lost severely and the image is white as a whole. RRDNet performed well on the original brighter images but performed relatively poorly on the darker images, where the brightness boost was not significant, making it difficult to achieve good visual effects. The LightenNet method showed average brightness improvement in the comparison method, but the image showed a white blocky phenomenon. Although Zero-DCE can retain the detail features of the image well, the brightness enhancement is not obvious, and the color contrast of the image is significantly reduced. The enhancement effect of DSLR produces obvious stacked block phenomenon, the whole wardrobe part appears as a block effect, and there are artifacts. For LLNet, it can be seen from the hanger and the enlarged part that details are seriously lost, and the image enhancement effect is blurred as a whole. Compared with other methods, the brightness improvement effect of the proposed method may not be the most ideal, but it effectively avoids other problems, such as color distortion, detail loss and artifact phenomenon, etc., and the brightness of the enhanced effect is improved as a whole without overexposure or underexposure phenomena. Figure 6 and Figure 7 are from the 5 K dataset. In Figure 6, the Retinex method has the highest visual brightness, but the overall image is overexposed and the visible details are seriously lost. The enhancement effect of the HE method is overall white. RRDNet, as with DSLR, has a low brightness boost, which has a big impact on visual effects.

### 5.2. Objective Evaluation

In addition to subjective visual evaluation, recognized image quality metrics for quantitative comparisons are used to illustrate the effectiveness of the algorithms in this paper. Many image quality evaluation metrics have been proposed in various image processing fields, of which *PSNR* [31] is the most widely used objective evaluation metric. *PSNR* stands for Peak Signal to Noise Ratio and is measured in dB, with larger values indicating less distortion. *SSIM* [32] is a measure of the similarity between two images. *SSIM* uses two images, one processed and the other real, to measure the similarity of the two images in terms of brightness, contrast, and structure, respectively. The value of *SSIM* is between 0 and 1, and the closer to 1 the higher the similarity. Both of these metrics have references, and for non-paired datasets, there are also non-reference metrics, such as *NIQE* [33], which tests the test images by extracting features from the natural landscape to more closely match the human eye’s visual perception. The *LOE* [34] reflects the natural holding power of the image, with smaller values indicating a better brightness order and a more natural look.

*PSNR* is used to evaluate the differences between images. It is widely used in image quality evaluation of low-level image processing tasks such as image de-fogging, image noise reduction, and image enhancement. The *PSNR* formula can be expressed as:

(7) $$PSNR=10\times \log\frac{MaxValue^{2}}{MSE}$$

where *MSE* is the mean square error between images, and *MaxValue* is the maximum pixel value of two images. The formula of *MSE* can be expressed as:

(8) $$MSE=\frac{1}{M\times N}\overset{M}{\underset{i=1}{\sum }}\overset{N}{\underset{j=1}{\sum }}{\left[g\left(x,y\right)-\hat{g}\left(x,y\right)\right]}^{2}$$

where *H* stands for image height and *W* stands for image width, and $g\left(x,y\right)$ and $\hat{g}\left(x,y\right)$ stand for the original image and enhanced image, respectively.

*SSIM* is used to highlight the brightness, contrast, and structural similarity between two images, and the value range is 0–1; the closer the value is to 1, the more similar the two images are. Assuming that *x* and *y* are two input images, the formula is:

(9) $$SSIM={\left[l\left(x,y\right)\right]}^{\alpha }{\left[C\left(x,y\right)\right]}^{\beta }{\left[S\left(x,y\right)\right]}^{\gamma }$$

where $l\left(x,y\right)$ is brightness comparison, $C\left(x,y\right)$ is contrast comparison, and $S\left(x,y\right)$ is structural comparison. α,  β,  γ are greater than 0, and are used to adjust the three-part specific gravity. $l\left(x,y\right)$, $C\left(x,y\right)$, and $S\left(x,y\right)$ have the following formulas, respectively:

(10) $$l\left(x,y\right)=\frac{2\mu_{x}\mu_{y}+c_{1}}{\mu_{x}{}^{2}+\mu_{y}{}^{2}+c_{1}}\;,\;C\left(x,y\right)=\frac{2\sigma_{xy}+c_{2}}{\sigma_{x}{}^{2}+\sigma_{y}{}^{2}+c_{2}}\;,S\left(x,y\right)=\frac{\sigma_{xy}+c_{3}}{\sigma_{x}\sigma_{y}+c_{3}}$$

where $\mu_{x}$ and $\mu_{y}$, respectively, represent the average values of the two images, $\sigma_{x}$ and $\sigma_{y}$ represent the standard deviations of the two images. $\sigma_{xy}$ represents the covariance of the two images. The function of $c_{1}$, $c_{2}$, and $c_{3}$ is to avoid the denominator being 0.

*LOE* is the sequential difference of brightness of an image, and the illumination change of an image is evaluated by evaluating the sequential change process of the brightness of the image in the neighborhood. *LOE* reflects the natural retention ability of the image. A smaller value indicates that the image has a better luminance order and looks more natural. The formula is:

(11) $$LOE=\frac{1}{M\times N}\overset{M}{\underset{i=1}{\sum }}\overset{N}{\underset{j=1}{\sum }}RD\left(i,j\right)$$

where $RD\left(i,\;j\right)$ is the difference in the relative brightness order between the original image and the enhanced image.

*NIQE* is based on a set of “quality-aware” features and fits them into the MVG model. The quality perception features are derived from a simple but highly regularized NSS model. Then, the *NIQE* index of a given test image is expressed as the distance between the MVG model of NSS features extracted from the test image and the MVG model of quality perception features extracted from the natural image corpus. The *NIQE* formula is:

(12) $$NIQE=D\left(v_{1},v_{2},m_{1},m_{2}\right)=\sqrt{({\left(v_{1}-v_{2}\right)}^{T}{\left(\frac{m_{1}+m_{2}}{2}\right)}^{-1}\left(v_{1}-v_{2}\right)})$$

where $v_{1}$,$\;v_{2}$,$\;m_{1}$, and $m_{2}$ represent the mean vector and covariance matrix of the natural MVG model and distorted image MVG model, respectively.

We evaluated the results of the proposed method and eight other representative methods on the *PSNR*, *SSIM*, *NIQE*, and *LOE* indicators on the LOL and 5 K datasets. It can be seen from Table 1 and Table 2 that no method can obtain the optimal value among all the image quality detection indicators. However, in the LOL dataset test, our method has the best performance on the *PSNR* index, and the second *SSIM* index is also better than most methods. In the test of the 5 K dataset, the optimal value of the *LOE* index was obtained, followed by *NIQE*, which also obtained the second place. Table 1 and Table 2 more strongly illustrate the effectiveness and applicability of the proposed approach.

## 6. Conclusions

In this paper, we propose a two-stage zero-shot low-illumination image enhancement network. Considering the mixed noise in low-illumination images and the loss of stylistic features by general methods, the model adopts a smoothing operation in the decomposition network to reduce image noise and obtain image texture feature images, which can effectively avoid the above two problems. Comparative experiments show that the proposed method is more consistent with human perception in the subjective visual angle, and performs well in the comparison of objective evaluation indicators.

## Figures and Tables

**Table 1:** The LOL dataset (paired dataset) was quantitatively evaluated according to *PSNR* and *SSIM*. The best results are shown in bold, the second is italic, and the third is underlined.

Method	PSNR↑	SSIM↑
Input	5.10	0.19
HE [10]	*15.23*	0.59
Retinex [19]	10.92	0.37
ExCNet [25]	14.32	0.75
RRDNet [27]	8.72	0.60
LightenNet [28]	7.87	0.52
Zero-DCE [29]	11.98	*0.76*
DSLR [30]	10.78	0.67
LLNet [16]	14.00	**0.78**
Ours	**17.84**	0.74

**Table 2:** The 5 K dataset (unpaired dataset) was quantitatively evaluated according to *NIQE* and *LOE*. The best results are shown in bold, the second is italic, and the third is underlined.

Method	NIQE↓	LOE↓
Input	28.12	0
HE [10]	30.76	254.87
Retinex [19]	23.33	291.14
ExCNet [25]	**17.96**	316.85
RRDNet [27]	18.47	*251.37*
LightenNet [28]	20.97	305.50
Zero-DCE [29]	21.50	351.37
DSLR [30]	18.40	272.58
LLNet [16]	26.35	302.76
Ours	*18.02*	**249.25**

## Data Availability

The data presented in this study are openly available at https://arxiv.org/abs/1808.04560 and https://data.csail.mit.edu/graphics/fivek/, accessed on 10 August 2018 and 2019, respectively.
